# The effect of regulating focus tendencies on college students’ sports anomie behavior: the mediating role of autonomous and controlled motivation

**DOI:** 10.3389/fpsyg.2025.1695619

**Published:** 2025-10-21

**Authors:** Yimeng Ma, Laibing Lu, Bin Gu, Jinfu Xu, Mingliang Chen

**Affiliations:** ^1^University Student Mental Health Education Guidance Center, Henan Medical University, Xinxiang, China; ^2^School of Physical Education and Sport Science, Fujian Normal University, Fuzhou, China; ^3^Basic Teaching and Research Department, Qingdao Engineering Vocational College, Qingdao, China; ^4^Department of Physical Education, Fujian Jiangxia University, Fuzhou, China; ^5^Fujian Provincial Institute of Sports Science, Fuzhou, China

**Keywords:** promotion focus, prevention focus, autonomous motivation, controlled motivation, sports anomie behavior

## Abstract

**Introduction:**

This study aimed to investigate the relationship between regulatory focus and sports anomie behavior among university students, examining the potential mediating roles of autonomous and controlled motivation.

**Methods:**

A survey was conducted with 2,128 university students using the Long-Term Regulatory Focus Scale, the Behavioral Regulation in Sport Questionnaire, and the College Students’ Sports Anomie Behavior Scale. The study examined the mediating roles of autonomous and controlled motivation in the relationship between regulatory focus and sports anomie behavior through structural equation modeling.

**Results:**

(1) promotion focus was significantly positively correlated with autonomous motivation, whereas prevention focus was significantly positively correlated with controlled motivation; (2) promotion focus and autonomous motivation were significant negative predictors of sports anomie behavior, while prevention focus and controlled motivation were significant positive predictors; and (3) autonomous motivation partially mediated the relationship between promotion focus and sports anomie behavior, and controlled motivation partially mediated the relationship between prevention focus and sports anomie behavior.

**Discussion:**

These findings highlight the distinct mediating mechanisms through which promotion focus and prevention focus influence sports anomie behavior, emphasizing the important roles of different motivational types in this process.

## 1 Introduction

Physical activity is widely recognized as a crucial means of promoting physical fitness and psychological well-being and has become an indispensable component of school education. For university students, participation in sports not only enhances physical health but also provides opportunities for developing teamwork skills, strengthening collective pride, and fostering well-rounded personal growth (Lou et al., 2024). However, with the increasing prevalence of sports activities, the phenomenon of sports anomie behavior among university students has garnered growing scholarly attention (Chen et al., 2023). Grounded in sociological anomie theory, this study defines “sports anomie behavior” as a systematic deviation from normative behavior exhibited by individuals in contexts such as physical education, training, or competition, resulting from insufficient internalization of sports ethics or the weakening of shared value standards. Typical manifestations include evading physical exercise, cheating on fitness tests, and intentionally violating competition rules (Liu et al., 2022). Conceptually, sports anomie behavior differs from the broader notion of “unethical behavior” by emphasizing the lack of normative identification within the specific domain of sports. It also differs from isolated “rule-breaking behavior,” as it reflects a more pervasive relational disorder between the individual and the sports normative system. Such behaviors not only compromise the fairness and educational value of sports activities but also negatively affect participants’ physical and mental health, moral development, and the overall campus sports culture (Yang et al., 2024). Consequently, a thorough investigation of the factors influencing sports anomie behavior and its underlying mechanisms holds significant theoretical and practical importance.

It is widely accepted in academia that sports anomie behavior is influenced by a combination of intrinsic psychological factors and extrinsic environmental factors (Sun et al., 2013). Previous studies have identified several key variables: external environmental factors include the organizational ethical climate (Yang et al., 2024), the clarity and enforcement of competition rules (Gong and Manly, 2023), and broader social norms (Gordon, 2018); internal psychological factors encompass an individual’s social identity (Engelberg and Moston, 2020), perfectionist tendencies (Andrew, 2025), and personal values (Holland and Slowiak, 2021). However, two significant theoretical gaps exist in the literature. First, there is a noticeable imbalance in current research, with an emphasis on external environmental factors and a relative neglect of deeper internal cognitive processes. Scholars have predominantly focused on the constraining role of external conditions while paying insufficient attention to the psychological mechanisms that drive individual decision-making. For example, little in-depth exploration has been conducted on how internal mental processes, such as the ways individuals perceive, interpret, and respond to their environment, or the “psychological representations” they form of others and their surroundings, mediate or moderate the impact of external factors on sports anomie behavior. Second, the generalizability of existing findings, which are largely based on studies of professional athletes, to the university student population remains uncertain. College students navigate a unique context characterized by academic pressures, identity formation, and complex peer relationships, which may give rise to distinct motivations underlying sports anomie behavior. Consequently, research specifically focused on this demographic remains scarce.

Regulatory focus theory (RFT; Higgins, 1997) provides a foundational framework for understanding individual differences in motivation and subsequent behavior. This theory distinguishes between two self-regulatory orientations: promotion focus, characterized by the pursuit of ideals and accomplishments with heightened sensitivity to positive outcomes, and prevention focus, which emphasizes safety, responsibility, and vigilance against negative outcomes (Chun and Zheng, 2022; Guo et al., 2024). This theoretical lens offers a powerful perspective for explaining university students’ decision-making processes in sports contexts. Evidence suggests that a promotion focus is associated with growth-oriented goals and rule-compliant behavior (Sáenz et al., 2013; Joseph and Prakash, 2025), whereas a prevention focus, driven by fear of failure, may increase the likelihood of engaging in improper conduct as a short-term risk-avoidance strategy (MacRae, 2025). Consequently, the prevailing discourse posits that promotion focus generally supports normative behavior, whereas prevention focus may elevate the risk of sports anomie behavior (Galen et al., 2024; Paratore, 2013). However, a critical gap remains in the application of RFT. Current research is largely speculative, lacking direct empirical evidence testing the causal relationships between these regulatory foci and sports anomie behavior among university students, including the relevant boundary conditions (Hou et al., 2024). More specifically, the underlying mediating mechanisms, such as how these motivational orientations influence behavioral decisions through cognitive-affective processes like risk perception or moral judgment, have yet to be clearly identified. Therefore, a primary direction for future research lies in the systematic empirical examination of RFT to elucidate its specific pathways in the development of sports anomie behavior within the student population.

Self-determination theory (SDT; Deci and Ryan, 1985) provides a foundational framework for understanding human motivation, conceptualizing it along a continuum from amotivation to intrinsic motivation. A key distinction within this framework lies between autonomous motivation, which includes identified regulation, integrated regulation, and intrinsic motivation, and controlled motivation, encompassing external regulation and introjected regulation (Ansteenkiste et al., 2008). This theoretical perspective is particularly valuable for elucidating the mechanisms linking regulatory focus to sports anomie behavior among university students. Theoretically, autonomous and controlled motivation are posited to mediate this relationship. A promotion focus, associated with the pursuit of the ideal self, is often driven by autonomous motivation, which may enhance rule identification and reduce sports anomie behavior (Udegbunam, 2024; Hoxha and Ramadani, 2024; Yakushina et al., 2024). Conversely, a prevention focus, oriented toward failure avoidance, is frequently associated with controlled motivation, whereby external pressures may increase the likelihood of rule-breaking behaviors (Eryücel et al., 2024; Talia and Audun, 2023; Sáenz et al., 2013). Integrating these motivational dimensions offers a promising avenue for revealing the psychological processes through which regulatory focus influences behavior (Khan et al., 2025; Bockorny et al., 2024).

Recent research has emphasized the importance of addressing psychological mechanisms such as motivation and fear in sports education. Studies indicate that technology-assisted interventions can help reduce psychological barriers and support students’ participation in physical education settings (Alkasasbeh et al., 2024). In addition, recent reviews have highlighted that intrinsic motivation has a stronger and more sustained effect on athletic performance compared to extrinsic motivation, which tends to produce short-term outcomes (Alkasasbeh and Akroush, 2025). Together, these findings underscore the need to investigate how motivational factors relate to behavioral outcomes, providing a solid foundation for examining the mediating role of autonomous and controlled motivation in sports contexts.

Despite extensive research on both SDT and RFT, their systematic integration to explain sports anomie behavior remains limited. In particular, empirical evidence is lacking on whether, and how, autonomous and controlled motivation serve as mediating pathways between regulatory focus and sports anomie behavior. Developing an integrated theoretical model to empirically test these motivational mediators would therefore provide a robust foundation for understanding the etiology of sports anomie behavior and for designing targeted interventions.

Based on the theoretical foundation outlined above, the following hypotheses are proposed: (1) Promotion focus will be associated with a reduction in sports anomie behavior, whereas prevention focus will be associated with an increase in such behavior. (2) Autonomous motivation will be linked to a greater reduction in sports anomie behavior compared to controlled motivation. (3) Promotion focus will be positively correlated with autonomous motivation and negatively correlated with controlled motivation, whereas prevention focus will be positively correlated with controlled motivation and negatively correlated with autonomous motivation. (4) Autonomous motivation will mediate the relationship between promotion focus and sports anomie behavior, while controlled motivation will mediate the relationship between prevention focus and sports anomie behavior.

## 2 Methods and materials

### 2.1 Research subjects

Data collection was conducted in March 2025. Using a cluster sampling method, 2,385 undergraduate students from six universities across different regions, including comprehensive, normal, and polytechnic institutions to ensure diversity in academic backgrounds, were selected as participants. A paper-based survey was uniformly administered, and all participants were adults. Prior to participation, informed consent was obtained from all respondents. To enhance data authenticity and minimize social desirability bias, participants were explicitly informed that the study was solely for academic purposes, assured of the anonymity and confidentiality of their responses, and told that no identifying information was required. Completed questionnaires were sealed by participants in provided envelopes before collection. Investigators emphasized that there were no right or wrong answers and encouraged participants to respond based on their actual experiences. The survey required approximately 15–20 min to complete. A total of 2,128 valid questionnaires were collected, yielding an effective response rate of 89.22%. The valid sample included 550 first-year students (26.3%), 557 s-year students (26.2%), 582 third-year students (27.3%), and 429 fourth-year students (20.2%). There were 1,111 male students (52.2%) and 1,017 female students (47.8%). Regarding geographical background, 907 participants (42.6%) were from urban areas and 1,221 (57.4%) were from rural areas. Information on participants’ major disciplines and annual household income was also collected to be used as control variables in subsequent analyses. This study was approved by the Ethics Committee of Henan Medical University (formerly Xinxiang Medical University; No. XYLL-20250458).

### 2.2 Research tools

#### 2.2.1 General long-term tendency adjustment focus scale

The Long-Term Regulatory Focus Scale (Lockwood et al., 2002) was administered to assess participants’ regulatory focus tendencies. This 18-item instrument comprises two 9-item subscales measuring promotion focus and prevention focus, respectively. Responses were recorded on a 5-point Likert scale ranging from 1 (strongly disagree) to 5 (strongly agree). The promotion focus subscale captures goal-setting orientations toward achievement (e.g., “I frequently think about how I will achieve my hopes and aspirations”), whereas the prevention focus subscale reflects orientations toward avoiding failure (e.g., “I often worry about how I can prevent failures in my life”). In the present study, internal consistency reliability coefficients were 0.78 for promotion focus and 0.70 for prevention focus. Confirmatory factor analysis indicated acceptable structural validity (*χ*^2^ = 212.41, *χ*^2^/df = 2.39, CFI = 0.92, TLI = 0.91, RMSEA = 0.084, SRMR = 0.056).

#### 2.2.2 Autonomous and controlled motivation

The Behavioral Regulation in Sport Questionnaire (Lonsdale et al., 2008) was used to assess participants’ motivation. This 20-item instrument comprises five subscales: intrinsic motivation (4 items, e.g., “I enjoy sports”), identified regulation (4 items, e.g., “The benefits of sports are important to me”), integrated regulation (4 items, e.g., “Sports are part of who I am”), introjected regulation (4 items, e.g., “I would feel ashamed if I quit”), and external regulation (4 items, e.g., “Others would be disappointed if I did not participate”). Responses were collected using a 7-point Likert scale ranging from 1 (strongly disagree) to 7 (strongly agree). Composite scores were calculated as follows: autonomous motivation = (2 × intrinsic motivation) + identified regulation + integrated regulation; controlled motivation = (2 × introjected regulation) + (2 × external regulation). The subscales demonstrated adequate internal consistency, with Cronbach’s *α* coefficients ranging from 0.76 to 0.84. Confirmatory factor analysis supported the measure’s structural validity (*χ*^2^/df = 3.27, RMSEA = 0.078, NFI = 0.91, IFI = 0.92, CFI = 0.91).

#### 2.2.3 Sports anomie behavior

The College Students’ Sports Anomie Behavior Scale (Chen et al., 2021) was used to assess the frequency of sports anomie behaviors among university students over the past year. This 20-item scale encompasses five dimensions: anomie behavior in physical education classes, in the national physical fitness standard test, in athletic competitions, in sports club activities, and in extracurricular self-directed exercise. Sample items include, “I have felt anxious or even resistant about my physical education exam scores.” Responses were recorded on a 5-point Likert scale. In the present study, Cronbach’s α coefficients for the five subscales ranged from 0.76 to 0.84. Confirmatory factor analysis indicated good structural validity of the scale (*χ*^2^/df = 3.27, RMSEA = 0.070, NFI = 0.90, IFI = 0.93, CFI = 0.93).

To ensure the validity and cultural appropriateness of the measurement instruments, all original English scales were translated and adapted following a standardized procedure. First, forward translation was independently performed by two PhDs in sports psychology, who translated the Long-Term Regulatory Focus Scale and the Behavioral Regulation in Sport Questionnaire into Chinese. The two versions were then compared and integrated into a preliminary Chinese version by a third researcher. Next, back-translation was conducted by two doctoral students in English literature who were unfamiliar with the original scales. The back-translated versions were compared with the original English scales by the research team, and no significant conceptual discrepancies were identified, confirming the semantic equivalence of the Chinese version. Subsequently, expert review and cultural adaptation were conducted by a panel of sports psychology professors and university physical education instructors. The panel evaluated content validity, conceptual relevance, and cultural appropriateness within the context of Chinese university sports. Minor revisions were made to the wording of certain items based on feedback to improve clarity and contextual fit. Finally, a pilot test was conducted using the revised Chinese questionnaire with 120 university students who were not included in the main study. Cognitive interviews ensured that all items were correctly understood, and the scales demonstrated good preliminary reliability, with Cronbach’s *α* values exceeding 0.75.

### 2.3 Statistical methods

Descriptive statistics, correlation analysis, reliability analysis, confirmatory factor analysis, and structural equation modeling were performed on the collected data using SPSS 22.0 and AMOS 17.0.

## 3 Results and analysis

### 3.1 Common method bias test

Common method bias was assessed using Harman’s single-factor test. All scale items were subjected to an unrotated principal component factor analysis. The results indicated that five factors had initial eigenvalues greater than 1, with the first factor accounting for 35.15% of the total variance, which is below the 40% threshold. Therefore, common method bias was not considered a serious concern in this study.

### 3.2 Descriptive statistics and correlation analysis

Paired-samples t-tests revealed that promotion focus scores were significantly higher than prevention focus scores (*t* = 12.324, *p* < 0.001), and autonomous motivation levels were significantly greater than controlled motivation levels (*t* = 53.847, *p* < 0.001). Pearson correlation analyses indicated significant positive associations between promotion focus and autonomous motivation (*r* = 0.416, *p* < 0.01), as well as between prevention focus and both controlled motivation (*r* = 0.260, *p* < 0.01) and sports anomie behavior (*r* = 0.376, *p* < 0.01). Significant negative correlations were observed between promotion focus and both prevention focus (*r* = −0.323, *p* < 0.01) and sports anomie behavior (*r* = −0.455, *p* < 0.01), and between autonomous motivation and sports anomie behavior (*r* = −0.330, *p* < 0.01). A positive correlation was also found between sports anomie behavior and controlled motivation (*r* = 0.310, *p* < 0.01). Detailed results are presented in Table 1. These findings support Hypotheses 1 and 2 and provide partial support for Hypothesis 3, establishing the necessary preliminary conditions for subsequent mediation analyses examining the roles of autonomous and controlled motivation in the relationship between regulatory focus and sports anomie behavior.

**Table 1:** Descriptive statistics and correlation analysis results (*n* = 2,128).

Variable	*M*	*SD*	1	2	3	4
1 Promotion focus	29.50	8.935	1			
2 Prevention focus	25.19	10.849	−0.323^**^			
3 Autonomous motivation	102.08	34.420	0.416^**^	−0.152		
4 Controlled motivation	57.55	19.144	−0.107	0.260^**^	0.073	
5 Sports anomie behavior	55.32	17.857	−0.455^**^	0.376^**^	−0.330^**^	0.310^**^

*P < 0.05, **P < 0.01, ***P < 0.001, the same applies below.

### 3.3 Testing the mediating role of autonomous and controlled motivation in regulating the influence of focus orientation on sports anomie behavior

**Figure 1:**
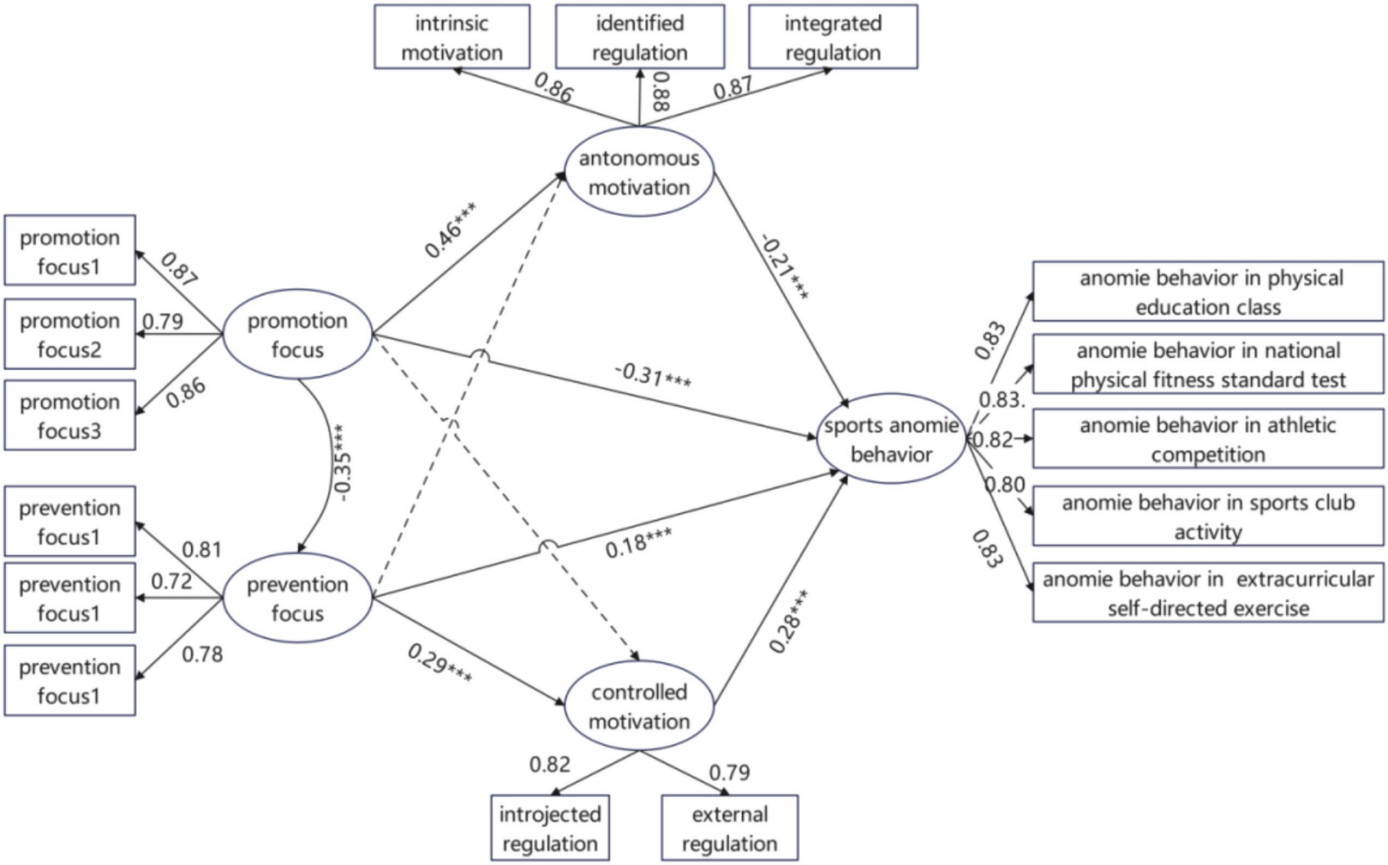
Mediating role of autonomous motivation and controlled motivation in the relationship between regulatory focus and sports anomie behavior.

The mediating roles of autonomous and controlled motivation in the relationship between regulatory focus and sports anomie behavior among university students were examined using structural equation modeling. A two-step analytical procedure, as proposed by Anderson and Gerbing (1988), was employed, first assessing the fit of the measurement model before proceeding to structural model analysis. Five latent variables were examined: promotion focus, prevention focus, autonomous motivation, controlled motivation, and sports anomie behavior. These were operationalized using the following observed indicators: three item parcels for promotion focus and three for prevention focus, created by bundling the first three, middle three, and final three items of each scale; the three subdimensions of autonomous motivation (intrinsic, identified, and integrated regulation) and the two subdimensions of controlled motivation (introjected and external regulation); and the five behavioral domains of sports anomie behavior (physical education class, national physical fitness standard test, athletic competition, sports club activity, and extracurricular self-directed exercise). The measurement model specification is illustrated in Figure 1. Confirmatory factor analysis results (Table 2) indicated acceptable model fit, supporting the structural validity of the measures.

**Table 2:** Overall fit coefficient.

Indicator	Reference standard	Actual test results
CMIN/DF	1–3 is excellent, 3–5 is Good	1.041
RMSEA	<0.05 is excellent, <0.08 is good	0.004
IFI	>0.9 is excellent, >0.8 is good	0.912
TLI	>0.9 is excellent, >0.8 is good	0.954
CFI	>0.9 is excellent, >0.8 is good	0.962
GFI	>0.9 is excellent, >0.8 is good	0.994
NFI	>0.9 is excellent, >0.8 is good	0.996

Path analysis was subsequently conducted using structural equation modeling to test the hypothesized relationships, with the results presented in Table 3. The core hypotheses of the model were largely supported. Distinct motivational activation pathways were observed for the two regulatory focus tendencies. Promotion focus exhibited a significant positive predictive effect on autonomous motivation (*β* = 0.461, *p* < 0.001), whereas prevention focus significantly and positively predicted controlled motivation (*β* = 0.287, *p* < 0.001). This pattern indicates that approach and avoidance tendencies correspond to distinct intrinsic and extrinsic motivational patterns, respectively. In contrast, the two cross-paths were non-significant (promotion focus → controlled motivation: *β* = −0.016, *p* = 0.540; prevention focus → autonomous motivation: *β* = 0.000, *p* = 0.993), demonstrating a high degree of specificity in how each regulatory focus type influences motivational pathways.

**Table 3:** Path parameter estimates of the structural equation model.

Path	*β*	Estimate	S. E.	C. R.	*P*	Assumption
Promotion focus → Autonomous motivation	0.461	1.769	0.095	18.590	***	establishment
Prevention focus → control motivation	0.287	0.749	0.067	11.239	***	establishment
Promotion focus → control motivation	−0.016	−0.051	0.084	−0.612	0.540	not established
Prevention focus → Autonomous motivation	0.000	0.001	0.070	0.009	0.993	not established
Promotion focus → sports anomie behavior	−0.308	−0.380	0.030	−12.596	***	establishment
Prevention focus → sports anomie behavior	0.180	0.177	0.021	8.409	***	establishment
Autonomous motivation → sports anomie behavior	−0.207	−0.066	0.007	−9.085	***	establishment
Control motivation → sports anomie behavior	0.276	0.104	0.009	11.781	***	establishment

In the predictive pathways for sports anomie behavior, autonomous motivation was a significant negative predictor (*β* = −0.207, *p* < 0.001), whereas controlled motivation significantly and positively predicted such behavior (*β* = 0.276, *p* < 0.001). After accounting for the mediating variables, promotion focus continued to exhibit a significant direct negative effect on sports anomie behavior (*β* = −0.308, *p* < 0.001), while prevention focus maintained a significant direct positive effect (*β* = 0.180, *p* < 0.001).

This framework illustrates how regulatory focus influences sports anomie behavior through motivational mediation, thereby providing a basis for subsequent mediation effect analyses.

Bootstrap test results, presented in Table 4, indicated a significant negative total effect of promotion focus on sports anomie behavior (*β* = −0.408). This total effect was decomposed into a significant direct effect (*β* = −0.308, *p* < 0.001) and a significant indirect effect. The indirect effect was estimated at −0.100, with a 95% confidence interval of [−0.125, −0.076], excluding zero, confirming that autonomous motivation partially mediated the relationship between promotion focus and sports anomie behavior. Conversely, prevention focus exhibited a significant positive total effect (*β* = 0.259), with both its direct effect (*β* = 0.180, *p* < 0.001) and indirect effect being statistically significant. The indirect effect was estimated at 0.079, with a 95% confidence interval of [0.062, 0.098], indicating that controlled motivation partially mediated the relationship between prevention focus and sports anomie behavior.

**Table 4:** Effect analysis of factors influencing sports anomie behavior.

Path	Effect	*β*	S. E.	*P*	95%CI	
					LB	UB
Promotion focus → Autonomous motivation → sports anomie behavior	Total effect	−0.408	0.021	0.000	−0.448	−0.367
	Direct effect	−0.308	0.026	0.000	−0.359	−0.258
	Indirect effect	−0.100	0.013	0.000	−0.125	−0.076
Prevention focus → control motivation → sports anomie behavior	Total effect	0.259	0.021	0.001	0.217	0.299
	Direct effect	0.180	0.021	0.000	0.137	0.221
	Indirect effect	0.079	0.009	0.000	0.062	0.098

In summary, regulatory focus exerted both direct effects on sports anomie behavior and indirect effects mediated through autonomous and controlled motivation, providing support for the core mediation hypotheses.

## 4 Discussion

### 4.1 Analysis of the characteristics and relationships between college students’ regulating focus tendencies, autonomous and controlled motivation, and sports anomie behavior

The results indicated that university students exhibited significantly higher levels of promotion focus than prevention focus. This phenomenon can be explained through a tripartite conceptual framework. From a developmental psychology perspective, the emerging adulthood stage is characterized by core developmental tasks such as identity exploration, future possibility expansion, and the pursuit of positive growth goals (e.g., academic and career advancement). This orientation toward an “ideal self” inherently reinforces a promotion focus (Wang et al., 2019). Environmental influences also contribute to this pattern (Ma and Zhao, 2023). The higher education system systematically cultivates a promotion focus through goal-oriented curriculum design, developmental assessment mechanisms emphasizing improvement, and an achievement-oriented culture, consistently channeling students’ attention toward attaining positive outcomes (Zou et al., 2023). Furthermore, cognitive neuroscience research provides corroborating evidence, indicating that the maturation of prefrontal cortex functionality during young adulthood enhances future scenario simulation and the valuation of positive outcomes, establishing a neurobiological basis for elevated promotion focus (Wang et al., 2011).

A significantly higher level of autonomous motivation relative to controlled motivation was also observed among university students, consistent with the findings of Lang et al. (2022). This motivational pattern can be primarily explained through the core mechanisms of SDT (Zhao et al., 2016). First, the university environment, characterized by flexible curriculum options, autonomous research practices, and personalized development pathways, effectively satisfies students’ basic psychological needs, particularly autonomy (e.g., academic decision-making) and competence (e.g., scaffolded skill development). This fulfillment of basic needs directly facilitates the emergence of intrinsic motivation. Second, with the maturation of formal operational thinking, university students demonstrate deeper internalization of learning goals (Yang and Zhang, 2016), enabling the integration of educational objectives such as knowledge acquisition and innovation capability into their self-concept (i.e., integrated regulation), rather than being primarily driven by external rewards or punishments. Furthermore, the ongoing transformation of higher education, evidenced by student-centered teaching models and formative assessment systems, fosters autonomy-supportive learning ecosystems (Zhao and Gao, 2017). These environments enhance the internalization of extrinsic motivation, allowing students to consistently experience a sense of self-determination during instructional interactions, thereby systematically elevating overall autonomous motivation (Zhang and Shen, 2005).

Correlational analyses revealed a significant negative relationship between promotion focus and sports anomie behavior among university students, whereas a significant positive relationship was observed between prevention focus and such behaviors. These associations can be interpreted through the core principles of RFT (Higgins, 1997). Individuals with a dominant promotion focus are oriented toward attaining positive outcomes, such as skill mastery and team achievement. Within sports contexts, this predisposition fosters rule compliance as a means of achieving growth-oriented goals. The pursuit of an “ideal self” strengthens achievement motivation and enhances self-regulatory capacity, thereby systematically reducing the likelihood of normative transgressions (Liu et al., 2024). In contrast, prevention-focused individuals primarily emphasize risk aversion, including avoiding defeat or negative evaluation. Under high-stakes competitive pressure, they may resort to extra-regulatory strategies, such as intentional fouls or prohibited substance use, as defensive measures against potential failure. This heightened sensitivity to obligations can trigger avoidant coping patterns (Chen, 2018), significantly increasing the propensity for preemptive anomie behaviors. Notably, these findings extend Hou’s (2024) theoretical framework from educational behavior to the sports domain, confirming the cross-contextual stability of regulatory focus in predicting behavioral transgressions. From an applied perspective, a promotion focus facilitates the internalization of sportsmanship values by activating intrinsic growth objectives, thereby promoting self-disciplined adherence to norms. Conversely, a prevention focus amplifies outcome-related anxiety, fostering instrumental motivations for rule-breaking that prioritize short-term risk avoidance over long-term normative identification. This fundamental divergence in motivational architecture underlies the opposing directional effects of the two regulatory foci on anomie behavior (Gong and Manly, 2023).

### 4.2 The mediating role of autonomous and controlled motivation in regulating the relationship between focus tendencies and sports anomie behavior

The mediating roles of autonomous and controlled motivation in the relationship between regulatory focus and sports anomie behavior were systematically examined using structural equation modeling with bootstrap sampling. The results confirmed that both types of motivation functioned as significant mediators; however, their underlying mechanisms followed distinct patterns, highlighting the critical role of motivational internalization in behavioral regulation.

Promotion focus exerted both a direct suppressive effect on sports anomie behavior and an indirect protective effect via the enhancement of autonomous motivation. This mechanism can be understood through the framework of motivational internalization and basic psychological needs. Oriented toward growth and development, promotion focus emphasizes ideal self-realization and accomplishment. This cognitive orientation facilitates greater recognition and integration of the intrinsic value of sports participation, thereby elevating autonomous motivation (Flavia and Diogenes, 2024). From the perspective of SDT, challenge-seeking contexts associated with a promotion focus align closely with individuals’ innate needs for competence and autonomy. This alignment promotes the internalization of external regulations, transforming them into personally endorsed values (Jakobsen, 2022). Consequently, students with high autonomous motivation are more likely to perceive sports participation as self-determined, cognitively accept rules, develop affective affiliation with sportsmanship, and demonstrate enhanced self-regulation and behavioral persistence. Thus, promotion focus reduces sports anomie behavior through a dual pathway: a direct effect and an indirect effect mediated by autonomous motivation. This pattern is consistent with SDT, which links intrinsic motivation and integrated regulation to positive behavioral outcomes, underscoring that behavior is more likely to align with social and ethical standards when psychological needs are satisfied and motivation is autonomous (Yan et al., 2025).

In contrast, prevention focus was found to operate through a partial mediation pathway via controlled motivation, a mechanism primarily rooted in external regulation and the activation of non-internalized behavioral motives. Emphasizing duty fulfillment and harm avoidance, prevention focus tends to trigger superficial compliance with external demands rather than genuine internalization, thereby heightening controlled motivation (Murphy and Steel, 2021). This motivational orientation is closely associated with external regulation (e.g., avoiding punishment) and introjected regulation (e.g., maintaining self-esteem or avoiding guilt), where behavior is driven by pressure or self-imposed coercion rather than authentic value endorsement. Students operating under such motivational conditions are more likely to engage in shortsighted, instrumental anomie behaviors, such as test cheating or impersonation, when confronted with stress, inadequate supervision, or high-stakes incentives, due to a lack of intrinsic behavioral regulation mechanisms (Yin et al., 2024). Analytical results confirmed that prevention focus not only directly predicted sports anomie behavior but also indirectly exacerbated it through increased controlled motivation. This pattern reveals a “dual-risk” mechanism in behavioral regulation associated with prevention focus: it directly activates anxiety and avoidance strategies, heightening sensitivity to failure and increasing the likelihood of behavioral transgressions, while simultaneously diminishing the self-determined quality of motivation and impeding the internalization of norms, making individuals more susceptible to deviating from standards when structural constraints are insufficient.

Based on these findings, universities and physical educators should establish supportive environments to mitigate sports anomie behaviors. For the promotion focus–autonomous motivation pathway, educational strategies should emphasize autonomy-supportive teaching practices. This can be achieved by incorporating student choice in activity selection, providing informational feedback focused on skill development, and designing cooperative learning tasks that minimize social comparison. Such approaches effectively enhance perceived autonomy and competence, thereby fostering autonomous motivation and intrinsically reducing the propensity for anomie behaviors. For the prevention focus–controlled motivation pathway, interventions should aim to reduce failure anxiety and external pressure. This can be accomplished by restructuring evaluation systems to emphasize effort and improvement rather than outcome-based punitive assessments. Additionally, through clear communication of rules and highlighting the intrinsic value of sports participation, external regulations can be internalized as personal values rather than adhered to out of fear of punishment. Collectively, these strategies target the underlying motivational mechanisms of sports anomie behavior and contribute to the development of healthier campus sports cultures.

## 5 Limitations and future prospects

This study has several limitations that warrant consideration. First, the generalizability of the findings is restricted, as the research was conducted within a single national and cultural context using a non-random sampling procedure. Second, all key variables were assessed via self-report measures, which are susceptible to response biases, particularly social desirability bias. For example, participants may have underreported engagement in sensitive behaviors such as sports anomie, potentially leading to an underestimation of the true relationships among variables. Third, the cross-sectional design raises the possibility of common method bias. Although procedural remedies, such as ensuring respondent anonymity, were implemented and statistical tests indicated that common method bias was not severe, future studies should adopt longitudinal or experimental designs to strengthen causal inference and incorporate multisource data to further mitigate this issue.

Future research could extend this work in several promising directions. First, examining potential moderators, such as sport type (individual vs. team) and institutional context (public vs. private universities), would help clarify boundary conditions and enhance the generalizability of the findings. Second, cross-cultural replications are needed to verify the robustness of the observed relationships across diverse populations. Methodological improvements could include employing more rigorous sampling designs, integrating multi-source data (e.g., peer ratings or behavioral records) to mitigate single-reporter bias, and utilizing indirect measurement techniques for sensitive constructs such as sports anomie behavior.

## 6 Conclusion

The key findings are as follows: Firstly, university students exhibited significantly higher levels of promotion focus than prevention focus, alongside stronger autonomous motivation compared to controlled motivation. Secondly, promotion focus was positively correlated with autonomous motivation, whereas prevention focus was associated with controlled motivation. Thirdly, promotion focus and autonomous motivation negatively predicted sports anomie behavior, while prevention focus and controlled motivation positively predicted such behavior. Finally, autonomous motivation partially mediated the relationship between promotion focus and sports anomie behavior, with controlled motivation similarly mediating the effect of prevention focus. Collectively, these findings underscore the importance of fostering promotion-oriented self-regulation and autonomous motivation in interventions aimed at mitigating sports anomie behavior, while also highlighting the need to address prevention-focused tendencies and externally driven motivators to promote ethical conduct in athletic contexts.

## Data Availability

The raw data supporting the conclusions of this article will be made available by the authors, without undue reservation.
